# *Helicobacter Pylori* infection in children with inflammatory bowel disease: a prospective multicenter study

**DOI:** 10.1186/s12887-024-04902-z

**Published:** 2024-06-29

**Authors:** Emanuele Dilaghi, Enrico Felici, Edith Lahner, Emanuela Pilozzi, Silvia Furio, Livia Lucchini, Giovanna Quatrale, Marisa Piccirillo, Pasquale Parisi, Sara Curto, Bruno Annibale, Alessandro Ferretti, Maurizio Mennini, Severino Persechino, Giovanni Di Nardo

**Affiliations:** 1 Department of Medical-Surgical Sciences and Translational Medicine, Sant’Andrea Teaching Hospital, Sapienza University of Rome, Rome, Italy; 2 Pediatric and Pediatric Emergency Unit, Children Hospital, AO SS Antonio E Biagio E C. Arrigo, Alessandria, Italy; 3 Department of Clinical and Molecular Medicine, Sant’Andrea Teaching Hospital, Sapienza University of Rome, Rome, Italy; 4 Department of Neurosciences, Mental Health and Sensory Organs (NESMOS), Faculty of Medicine and Psychology, Sapienza University of Rome, Pediatric Unit, Sant’Andrea University Hospital, Via Grottarossa 1035, Rome, 00189 Italy

**Keywords:** *Helicobacter pylori* infection, *H. pylori* gastritis, Inflammatory Bowel Disease, Children

## Abstract

**Background:**

The relationship between *Helicobacter-pylori*(Hp)infection and inflammatory-bowel-disease(IBD) in pediatric-patients remains controversial. We aimed to assess the Hp-infection occurrence in newly-diagnosed pediatric-patients with IBD compared to no-IBD patients. Additionally, we aimed to examine differences in clinical-activity-index(CAI) and endoscopic-severity-score(ESS)between IBD-patients with and without Hp-infection, at baseline and at 1-year-follow-up(FU), after eradication-therapy(ET).

**Methods:**

IBD diagnosis was based on Porto-criteria, and all patients underwent gastroscopy at baseline and 1-year FU. For Crohn's-disease(CD) and ulcerative colitis(UC), IBD-CAI and -ESS were classified using PCDAI/SES-CD and PUCAI/UCEIS, respectively.

**Results:**

76 IBD-patients were included in the study[35 F(46.1%),median-age 12(range 2–17)]. CD and UC were diagnosed in 29(38.2%) and 45(59.2%)patients, respectively, and unclassified-IBD in two(2.6%)patients. Non-IBD patients were 148[71 F(48.0%),median-age 12(range 1–17)]. Hp-infection at baseline was reported in 7(9.2%) and 18(12.2%)IBD and non-IBD patients, respectively(*p* = 0.5065).

The 7 IBD patients with Hp infection were compared to 69 IBD patients without Hp-infection at baseline evaluation, and no significant differences were reported considering CAI and ESS in these two groups.

At 1-year FU, after ET, IBD patients with Hp infection improved, both for CAI and ESS, but statistical significance was not reached.

**Conclusion:**

The occurrence of Hp-infection did not differ between IBD and no-IBD patients. No differences in CAI or ESS were observed at the diagnosis, and after ET no worsening of CAI or ESS was noted at one-year FU, between Hp-positive and -negative IBD patients.

**Supplementary Information:**

The online version contains supplementary material available at 10.1186/s12887-024-04902-z.

## Background

Epidemiological evidence indicates that *Helicobacter pylori* (*H. pylori*) infection is less prevalent in patients with inflammatory bowel disease (IBD) compared with healthy controls. Data from a recent meta-analysis support the hypothesis that *H. pylori* infection may have a protective role against IBD development [1]. The finding that the prevalence of IBD has been increasing in areas with lower rates of *H. pylori* colonization, such as the United States, seems to endorse this concept [2].

Data in pediatric populations are mainly based on retrospective studies with limited sample sizes. The largest pediatric retrospective study confirmed findings from adult studies showing that *H. pylori* gastritis is less prevalent in children with IBD than controls, indicating an inverse association between *H. pylori* and IBD [3]. Prospective large cohort studies are needed to distinguish between a true protective role of *H. pylori* and a confounding effect due to previous antibiotic use in children with IBD.

Moreover, it has been reported that IBD may occur or worsen after *H. pylori* eradication both in adult [3–5] and pediatric patients [6]. Nevertheless, the impact of *H. pylori* eradication on the incidence of IBD and its effect on the activity of IBD may remain uncertain.

Our prospective study aimed to assess: 1) the occurrence of *H. pylori* gastritis in newly diagnosed pediatric patients with IBD, in comparison to non-IBD children undergoing upper GI endoscopy; 2) possible differences in the clinical phenotype between IBD patients with and without *H. pylori* infection; 3) impact of *H. Pylori* eradication on IBD outcome at one-year follow-up.

## Methods

This article has been drafted following the STROBE guidelines, ensuring the quality of reporting [7].

### Working definition

**Fig. 1:**
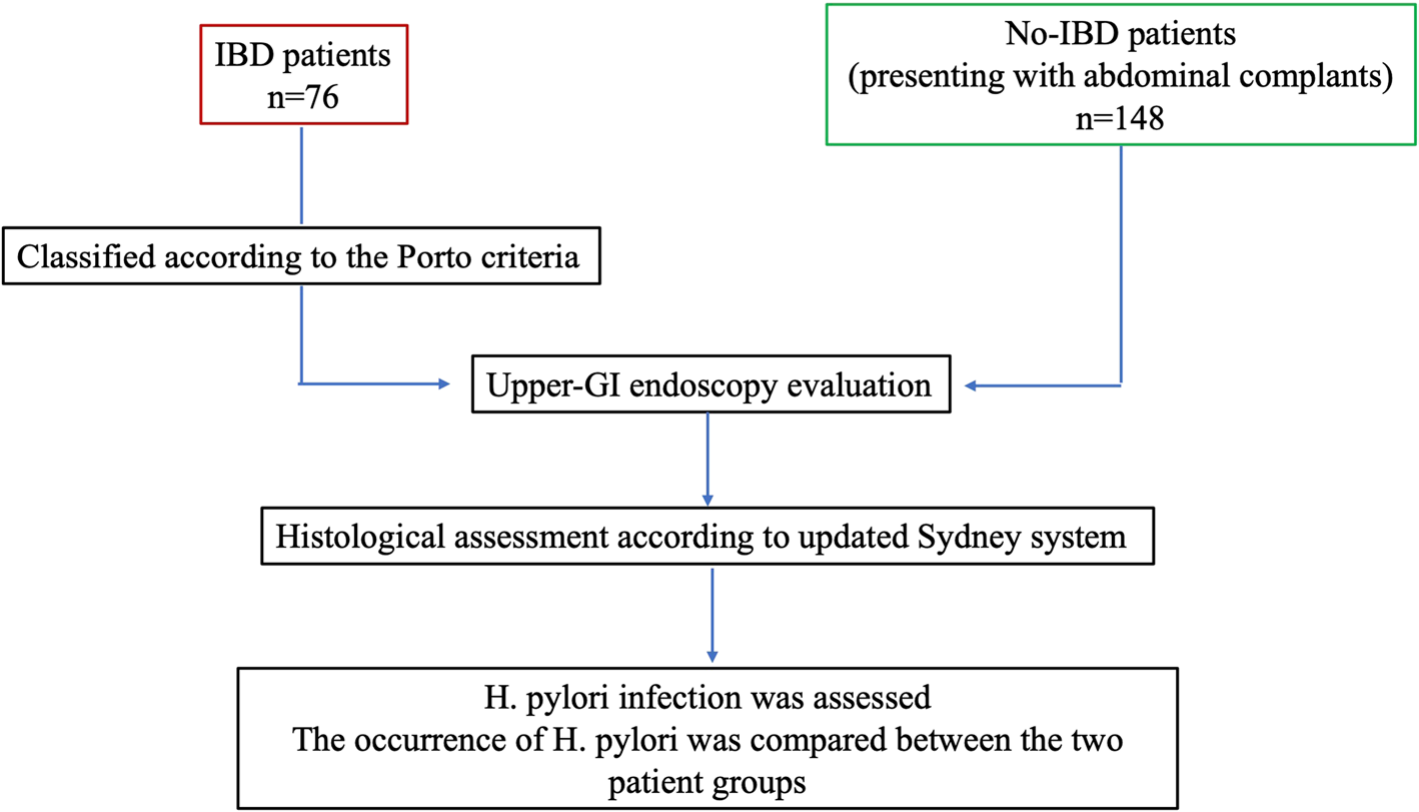
Flowchart of the study population, regarding the *H. pylori* prevalence among inflammatory bowel disease (IBD) patients and no-IBD patients

This study compared the *H. pylori* occurrence between IBD pediatric patients and no-IBD patients (Fig. 1). Patients in the first group underwent lower and upper-GI endoscopy evaluation to complete the diagnostic work-up. The no-IBD patients were pediatric patients referred for abdominal complaints undergoing gastroscopy, considered necessary to complete the clinical assessment.

All patients diagnosed with *H. pylori* infection underwent eradication therapy, according to a specific therapeutic regimen, reported below.

To define the eventual presence of clinical differences at the diagnosis among IBD patients, two sub-groups were defined according to the presence of *H. pylori* infection: IBD patients with *H. pylori* infection and IBD patients without *H. pylori* infection. These two sub-groups were compared considering the clinical and endoscopic activity of IBD patients, according to the Pediatric Crohn’s Disease Activity Index/Simple Endoscopic Score for Crohn’s Disease (PCDAI/SES-CD) and the Pediatric Ulcerative Colitis Activity Index/Ulcerative Colitis Endoscopic Index of Severity (PUCAI/UCEIS) for Crohn’s disease and Ulcerative colitis, respectively.

Lastly, to define the eventual clinical impact of *H. pylori* eradication in IBD patients, we compared the two sub-groups considering the same parameters, as previously described, at one-year follow-up with clinical and upper/lower GI endoscopy evaluation.

### Study population

This prospective study was conducted across two participating centers: the Pediatric Gastroenterology Unit of Sapienza—University of Rome and the Pediatric Gastroenterology Unit of “C. Arrigo” Children’s Hospital of Alessandria, Italy. Comprehensive data were collected from all patients (2019–2022), encompassing clinical information, endoscopic findings, gastric biopsy specimens, and microscopic analyses. All patients were newly diagnosed with IBD based on the Porto criteria [8] and further classified into: Crohn’s Disease (CD), ulcerative colitis (UC), and IBD unclassified (IBDU).

At the time of diagnosis of IBD, all patients underwent upper GI endoscopy to complete the diagnostic work-up. The following sampling protocol was applied: second part of duodenum (4 biopsies), antrum (3 biopsies), body (3 biopsies), fundus (3 biopsies), and esophagus (2**–**4 biopsies). All IBD patients underwent regular clinical follow-up, blood tests every 3 months, and fecal calprotectin assessments every 6 months. They also underwent upper and lower GI endoscopy evaluation at a one-year follow-up.

The no-IBD group consisted of patients who underwent initial upper GI endoscopy to investigate abdominal complaints.

Children undergoing upper GI endoscopy for iron-deficiency anaemia of unknown origin were excluded [3].

Among the patients with *H. pylori* infection, after undergoing eradication therapy, all underwent a one-year follow-up upper-GI evaluation to assess the success of *H. pylori* eradication.

For children with histologically assessed gastritis without detectable *H. pylori*, a urea breath test (UBT) was performed. The UBT was performed at least one month after discontinuing medications such as antibiotics, proton pump inhibitors, or H2 antagonists that had been eventually administered before the endoscopy.

The protocol received approval from the hospital's ethics committee, and the study was conducted in adherence to the principles outlined in the Declaration of Helsinki.

### Sampling protocol and patients’ classification

Trained pediatric gastroenterologists (GDN and SF) performed endoscopies, and the following biopsies were taken in all cases: the second part of the duodenum (4 biopsies), antrum (3 biopsies), body (3 biopsies), fundus (3 biopsies) and esophagus (2**–**4 biopsies). Specimens were fixed in 10% formalin, and embedded in paraffin, and sections were stained with hematoxylin–eosin and modified Giemsa or Masson trichrome and assessed under light microscopy. All histopathological analyses were performed by a highly experienced histopathologist (EP).

IBD patients' clinical and endoscopic activity were classified using the PCDAI/SES-CD and the PUCAI/UCEIS for Crohn’s disease and Ulcerative colitis, respectively [9–11]. IBD therapy was based on published guidelines [12, 13]. The eventual presence of chronic inflammation (e.g. chronic gastritis) was classified according to the updated Sydney system [14]. Bacterial density and gastric inflammation were scored according to the updated Sydney system. *H. pylori* infection was defined according to published guidelines [15].

### *H. pylori* eradication protocol

*H. pylori* eradication was performed using the sequential protocol (omeprazole plus amoxicillin for 5 days, followed by omeprazole plus clarithromycin plus tinidazole for another 5 days) [16].

### Case–control sub-analysis

The case–control model was used to eventually confirm results obtained from the analysis of the whole cohort. Cases were defined as IBD patients with *H. pylori* infection, and were matched according to gender, age at the diagnosis (± 2 years), and type of IBD, according to a 1:3 ratio with controls, defined as IBD patients with no evidence of *H. pylori* infection.

### Statistical analyses

Data were presented as mean ± standard deviation for continuous variables or absolute (n) and relative (%) frequencies for categorical variables. Means were compared by Student’s t-test and proportions by Fisher’s exact test. A *p*-value ≤ 0.05 was considered statistically significant. By considering a prevalence of 3.8% [17] of *H. pylori* infection in IBD children and a sample size of 64 patients, a type I error-alpha of 0.1 and a type II error-beta of 0.2 was calculated. Statistical analyses were performed by using MedCalc Statistical Software version 20.113 (MedCalc Software, Ostend, Belgium; http://www.medcalc.org; 2022).

## Results

The study included a total of 76 patients newly diagnosed with IBD, of whom 35 (46.1%) were female. The median age at the diagnosis was 12 (range 2–17) years. Among these patients, 29 (38.2%) were diagnosed with Crohn’s disease (CD), 45 (59.2%) with ulcerative colitis (UC), and 2 (2.6%) were classified as having unclassified IBD. The no-IBD group consisted of 148 patients, 71 (48.0%) females, and the median age was 12 (range 1–17) years.

### Occurrence of *H. pylori* infection in IBD compared to non-IBD pediatric patients

**Fig. 2:**
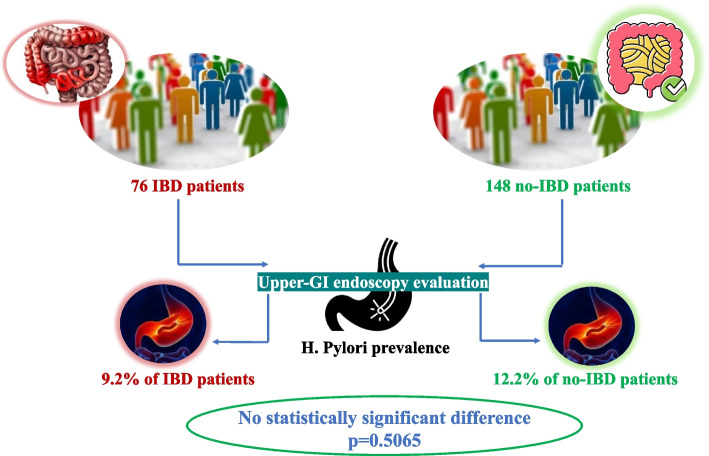
Comparison of *H. pylori* prevalence between inflammatory bowel disease (IBD) and no-IBD pediatric patients

*H. pylori* infection at baseline was reported in 7 (9.2%) out of 76 IBD patients compared to 18 (12.2) out of 148 non-IBD patients (*p* = 0.5065) (Fig. 2).

Regarding the clinical presentation in non-IBD patients with *H. pylori* infection, the most prevalent symptom was dyspepsia/epigastric pain, reported in 44.4% of patients (Please, see Additional file 1-Supplemental Table 1).

### Comparison between IBD patients with and without *H. pylori* infection

As reported above, *H. pylori* infection was diagnosed in 7 IBD patients, and these patients were compared to 69 IBD patients without *H. pylori* infection at baseline. Females were 28.6% and 47.8% (*p* = 0.4415), and the median age was 14 (range 12–16) years and 12 (range 2–17) years (*p* = 0.0391), in *H. pylori-infected* IBD patients and in IBD patients without *H. pylori* infection, respectively. UC was diagnosed in 57.1% and 59.4% of IBD patients (*p* = 1.000), with and without *H. pylori* infection, respectively. CD was reported in 42.9% of IBD patients with *H. pylori* infection, and in 37.7% of IBD patients without *H. pylori* infection (*p* = 1.000). Table 1 shows no significant differences considering the clinical activity index and endoscopic severity scores, both in CD and UC patients.

**Table 1:** Comparison between inflammatory bowel diseases (IBD) patients with and without *H. pylori* infection at baseline

	**IBD patients with *H. pylori* infection** ***n* = 7**	**IBD patients without *H. pylori* infection** ***n* = 69**	***p***
Female	2 (28.6)	33 (47.8)	0.4415
Median age, years (range)	14 (12–16)	12 (2–17)	0.0391
**Ulcerative colitis**	4 (57.1)	41 (59.4)	1.000
PUCAI 0—35	2 (50.0)	22 (53.7)	1.000
PUCAI > 35	2 (50.0)	19 (46.3)	1.000
UCEIS 0—4	3 (75.0)	17 (41.5)	0.3087
UCEIS 5—8	1 (25.0)	24 (58.5)	0.3087
**Crohn disease**	3 (42.9)	26 (37.7)	1.000
PCDAI 0—40	3 (100.0)	20 (76.9)	1.000
PCDAI > 40	0	6 (23.1)	1.000
SES-CD 0—6	0	4 (15.4)	1.000
SES-CD > 7	3 (100.0)	22 (84.6)	1.000

Data were expressed as number (percentage) of the total

*PUCAI* Pediatric Ulcerative Colitis Activity Index

*UCEIS* Ulcerative Colitis Endoscopic Index of Severity

*PCDAI* Pediatric Crohn’s Disease Activity Index

*SES-CD* Simple Endoscopic Score for Crohn’s Disease

### Case–control comparison between IBD patients with *H. pylori* infection (cases) and without *H. pylori* infection (controls)

Seven IBD patients with *H. pylori* infection (cases) were age-, gender-, and IBD-type-matched at a 1:3 ratio with 21 IBD patients without *H. pylori* infection. As reported in the Additional file 2-Supplemental Table 2, no significant differences were reported regarding activity index and endoscopic severity scores, both for CD and UC.

### Comparison between baseline and follow-up activity index and endoscopic severity scores in IBD patients

We observed a general improvement in CD and UC at follow-up, considering both clinical activity index and endoscopic severity scores. Table 2 shows a significant increase in patients with low activity index and endoscopic severity scores, and a significant reduction in patients with higher activity and endoscopic severity scores was reported. At follow-up, IBD patients who were successfully cured of *H. pylori* infection also showed improvement in both activity index and endoscopic severity scores, although statistical significance was not achieved.

**Table 2:** Comparison between baseline and follow-up activity scores in all patients with inflammatory bowel diseases (IBD) and those with and without *H. pylori* infection

	**Baseline**	**Follow-up**	***p***
Total IBD patients *n* = 76			
Ulcerative colitis *n* = 45 (59.2)			
PUCAI 0—35	24 (53.3)	40 (88.9)	0.0004
PUCAI > 35	21 (46.7)	5 (11.1)	0.0004
UCEIS 0 – 4	20 (44.4)	38 (84.4)	0.0001
UCEIS 5 – 8	25 (55.6)	7 (15.6)	0.0001
Crohn disease *n*= 29 (38.2)			
PCDAI 0—40	23 (79.3)	29 (100.0)	0.0235
PCDAI > 40	6 (20.7)	0	0.0235
SES-CD 0—6	4 (13.8)	18 (62.1)	0.0003
SES-CD > 7	25 (86.2)	11 (37.9)	0.0003
IBD patients with *H. pylori* infection *n* = 7			
Ulcerative colitis *n* = 4 (57.1)			
PUCAI 0 – 35	2 (50.0)	4 (100.0)	0.4286
PUCAI > 35	2 (50.0)	0	0.4286
UCEIS 0—4	3 (75.0)	4 (100.0)	1.000
UCEIS 5—8	1 (25.0)	0	1.000
Crohn disease *n* = 3 (42.9)			
PCDAI 0—40	3 (100.0)	3 (100.0)	1.000
PCDAI > 40	0	0	1.000
SES-CD 0—6	0	3 (100.0)	1.000
SES-CD > 7	3 (100.0)	0	1.000
IBD patients without *H. pylori* infection *n* = 69			
Ulcerative colitis *n* = 41 (59.4)			
PUCAI 0 – 35	22 (53.7)	36 (87.8)	0.0013
PUCAI > 35	19 (46.3)	5 (12.2)	0.0013
UCEIS 0—4	17 (41.5)	34 (82.9)	0.0002
UCEIS 5—8	24 (58.5)	7 (17.1)	0.0002
Crohn disease *n* = 26 (37.7)			
PCDAI 0—40	20 (76.9)	26 (100.0)	0.0226
PCDAI > 40	6 (23.1)	0	0.0226
SES-CD 0—6	4 (15.4)	15 (57.7)	0.0034
SES-CD > 7	22 (84.6)	11 (42.3)	0.0034

Data were expressed as number (percentage) of the total

*PUCAI* Pediatric Ulcerative Colitis Activity Index

*UCEIS* Ulcerative Colitis Endoscopic Index of Severity

*PCDAI* Pediatric Crohn’s Disease Activity Index

*SES-CD* Simple Endoscopic Score for Crohn’s Disease

As reported in Table 3, at follow-up, the activity index and endoscopic severity scores of CD and UC patients were similar to those at baseline, and this was also observed in the case–control model (Please, see Additional file 3-Supplemental Table 3).

**Table 3:** Comparison of activity scores between inflammatory bowel disease (IBD) patients with and without *H. pylori* infection at follow-up

	IBD patients with *H. pylori* infection *n* = 7	IBD patients without *H. pylori* infection *n* = 69	***p***
Female	2 (28.6)	33 (47.8)	0.4415
Median age, years (range)	14 (12–16)	12 (2–17)	0.0391
**Ulcerative colitis**	4 (57.1)	41 (59.4)	-
PUCAI 0—35	4 (100.0)	36 (87.8)	1.000
PUCAI > 35	0	5 (12.2)	1.000
UCEIS 0—4	4 (100.0)	34 (82.9)	1.000
UCEIS 5—8	0	7 (17.1)	1.000
**Crohn disease**	3 (42.9)	26 (37.7)	-
PCDAI 0—40	3 (100.0)	26 (100.0)	1.000
PCDAI > 40	0	0	1.000
SES-CD 0—6	3 (100.0)	15 (57.7)	0.2685
SES-CD > 7	0	11 (42.3)	0.2685

Data were expressed as number (percentage) of the total

*PUCAI* Pediatric Ulcerative Colitis Activity Index

*UCEIS* Ulcerative Colitis Endoscopic Index of Severity

*PCDAI* Pediatric Crohn’s Disease Activity Index

*SES-CD* Simple Endoscopic Score for Crohn’s Disease

## Discussion

The relationship between *H. pylori* and IBD is still controversial. Currently, several cross-sectional and retrospective studies reported in adult patients an inverse relation between *H. pylori* infection and IBD [18–21], but data in the pediatric population are scanty.

To our best knowledge, this is the first prospective multicenter study on a pediatric population aiming to define the occurrence of *H. pylori* infection in patients with IBD diagnosis compared to a pediatric population without IBD undergoing gastroscopy for upper GI symptoms, to explore the eventual occurrence of clinical differences between IBD patient groups, concerning the presence or absence of *H. pylori* infection, and to assess the clinical activity index and endoscopic severity scores of IBD patients at one-year follow-up, after *H. pylori* eradication.

In this study, no difference was observed (*p* = 0.5065) between the occurrence of *H. pylori* infection between IBD patients (9.2%) and healthy controls (12.2%).

This result contrasts with those observed in previous studies on adult patients. Parente et al. [18] reported an overall seroprevalence of *H. pylori* infection of 48% in IBD patients vs 59% in the control group (*p* < 0.05), thus showing a significantly lower frequency in CD vs UC patients. Ando et al. [19], in 2008, reported a similar result, as *H. pylori* infection (defined using the ^13^C-urea breath test) was significantly more frequent in controls than in CD patients (42% vs 8%, respectively). Again, more recently, Ali et al. [20], reported an overall positivity of *H. pylori* infection (defined by stool antigen test) of 14.3% in UC patients, significantly lower than 41.9% in the gender-age matched control group. Another case–control study by Song et al. [21], reported an *H. pylori* infection prevalence (defined by urea breath test) of 25.3% in IBD patients compared to 52.5% in healthy controls (*p* < 0.001). This association was more evident in patients younger than 60 years of age, indicating that *H. pylori* infection might be deemed to lower the possible risks of IBD in younger adults. Data on the prevalence of *H. pylori* infection in younger IBD patients are lacking. In 2014, Roka et al. [17] aimed to retrospectively assess *H. pylori* infection in a large number of newly diagnosed, treatment naïve children with IBD (mean age 7.3 years) and non-IBD children (mean age 9.3 years) undergoing upper GI endoscopy. The prevalence of *H. pylori* positivity was 3.8% in IBD and 13.2% in controls, and logistic regression showed that *H. pylori*-negative patients were 4.8-fold more likely to belong to the IBD group than *H. pylori*-positive patients (*p* < 0.001).

This data should be contextualized. Many published studies have reported a correlation between *H. pylori* infection and a lowered risk of developing extra-gastric diseases, such as gastroesophageal reflux disease, esophagitis, asthma, IBD, and other autoimmune diseases [22]. Defining the concept of the “hygiene hypothesis”, first introduced by David Strachan, today is also known as the “Old Friends Hypothesis” could be the key to interpreting this data. Modernization has reduced access to many of the immunoregulatory stimuli, these “Old Friends”, such as intestinal parasites, ectoparasites, gut commensal organisms, and also *H. pylori* [23–28] that humans have co-evolved with [23].

A recent meta-analysis [29] focused on the prevalence of *H. pylori* infection worldwide and specifically analyzed this prevalence by country. In Italy, a prevalence of 20.9% (13.0 – 31.9) was observed, quite different from that observed in our study in patients without a diagnosis of IBD. We think that this difference could be due to several factors. The types of specific studies considered in the meta-analysis above; the prevalence could be affected by the different methods used to detect *H. pylori* infection, and it could also depend on the not large cohort of patients included in our study.

Several mechanisms may be implicated in an eventual *H. pylori*-mediated protection against IBD. One possibility is that *H. pylori* infection and its eventual eradication may modify the gut microflora and immune responses eventually resulting in reduced intestinal inflammatory response which is thought to be one key factor of IBD development. [30]. Higher amounts of IL-10-secreting T-regulatory cells (Tregs) were shown to be present in the peripheral blood of *H. pylori*-infected patients [31]. IL-10 is a well-known anti-inflammatory and immunomodulatory cytokine, and Tregs are considered a mainstay in maintaining persistent *H. pylori* colonization, via suppression of proactive immunity. IBD patients (both CD and UC) tend to exhibit a marked deficiency in Tregs during relapses [32–34]. Papamichael et al. [35] showed that *H. pylori* infection may play a role against IBD by increasing the levels of some cytokines, activating dendritic cells and T cells, downregulating the Th1/Th17 pathway, and increasing Treg cell immune response.

Some meta-analyses including studies mainly conducted on adults, seem to confirm the inverse relationship between *H. pylori* and IBD development. Luther et al. [36] performed a meta-analysis on 5903 patients showing a pooled relative risk of *H. pylori* infection of 0.64 (95%CI 0.54–0.75), in IBD patients; a significant heterogeneity in the included studies was reported (I^2^ = 75.8%). Wu et al. [37] and Rokkas et al. [38] reported a pooled relative risk of 0.48(95%CI 0.43–0.54) and 0.62(95%CI 0.55–0.71), of *H. pylori* infection in IBD patients, respectively. In the meta-analysis by Wu et al. [36], no significant heterogeneity in the included studies was observed (I^2^ = 21%); on the contrary, in the meta-analysis by Rokkas et al. [38], there was considerable heterogeneity in the included studies (I^2^ = 77%). More recently, Shirzard-Aski et al. [1], including 58 studies with 13.549 IBD patients and 506.554 controls, reported a significant negative association between *H. pylori* infection and IBD (pooled OR 0.45, 95%CI 0.39–0.53, *p* < 0.001). Significant heterogeneity in the included studies was reported (I^2^ = 79%). Thus, these data provide evidence of a protective benefit of *H. pylori* infection against the development of IBD. Unfortunately, most of the evidence shown from these studies are weak, contradictory, and/or inconclusive. This tends to be because of constraints in study design; most studies are cross-sectional, comparing the seroprevalence of *H. pylori* in groups with and without disease. Wide variations in population size, in the test used for defining *H. pylori* infection, and demographic differences between studies mean many conflicting reports, resulting in a significant heterogeneity among the included studies.

In the current study, IBD patients were clinically and endoscopically evaluated at one-year follow-up, after *H. pylori* eradication. No worsening in the clinical activity index and endoscopic scores were observed between IBD patients with and without *H. pylori* infection. In 2001, Jovanovic et al. [4] reported that a patient developed Crohn’s disease three months after *H. pylori* eradication therapy was prescribed due to dyspeptic symptoms. Tursi [3] reported on two further patients who developed CD after receiving eradication therapy for *H. pylori.* Fujita et al. [6], reported that in a 12-year-old boy with UC in remission, after receiving eradication therapy for *H. pylori* infection, UC relapsed. These case reports are in contrast with our findings, and those results could be considered as a casual association instead of being attributed to a causal association. No other prospective studies conducted on a pediatric population are available.

Our study suffers from some limits. It should be noted that the prevalence of *H. pylori* infection in the non-IBD group might be overestimated because the patients reported abdominal symptoms. Even though the number of patients with an IBD diagnosis seems to meet the appropriate sample size, we are aware that our results do not come from a large cohort.

No relational causality to the relation between *H. pylori* and IBD is possible to obtain*:* more studies, possibly developing basic molecular mechanisms, are needed to elucidate this correlation further. On the contrary, our study seems to be acquiring strength, considering that the patients included come from a pediatric population, all of them were newly diagnosed and followed up for one year, and every patient underwent upper-GI endoscopy with biopsies both at baseline and after one year for detecting infection/eradication of *H. pylori.* The multicenter nature permitted the obtaining of more reproducible data. The case–control sub-analysis allowed for a uniform the comparison between sample-population considered and consequently the results obtained. Histopathological evaluation was centralized, as a single, highly experienced histopathologist performed all histopathological analyses.

In conclusion, the occurrence of *H. pylori* infection did not differ between IBD and no-IBD pediatric patients undergoing gastroscopy for upper GI symptoms, nor did it adopt a case–control-based comparison. No significant differences in clinical activity index and endoscopic scores were observed at the diagnosis among IBD patients with and without *H. pylori* infection. After *H. pylori* eradication, no worsening of clinical activity index or endoscopic scores were noted at one-year follow-up.

### Supplementary Information

Additional file 1:  Supplemental Table  1. Baseline characteristics of no-IBD patients with *H. pylori* infection.

Additional file 2:  Supplemental Table  2. Case–control model-based comparison between inflammatory bowel diseases (IBD) patients with H. pylori infection (cases) and without *H. pylori* infection (controls) at baseline. Cases and controls were matched for age at diagnosis (± 2 years), gender, and type of IBD (Crohn's disease or ulcerative colitis).

Additional file 3:  Supplemental Table  3. Case–control model-based comparison between inflammatory bowel diseases (IBD) patients with *H. pylori* infection (cases) and without *H. pylori* infection (controls) at follow-up. Cases and controls were matched for age at diagnosis (± 2 years), gender, and type of IBD (Crohn’s disease or ulcerative colitis).

## Data Availability

The datasets generated during and/or analysed during the current study are available from the corresponding author on reasonable request.
