# Supplementary material for: Helicobacter Pylori infection in children with inflammatory bowel disease: a prospective multicenter study
Source: BMC Pediatr. 2024 Jun 29;24:417. doi: 10.1186/s12887-024-04902-z (PMC11218114; doi:10.1186/s12887-024-04902-z)
Supplement: Supplementary file 1 — Additional file 1: Supplemental Table 1. Baseline characteristics of no-IBD patients with H. pylori infection. [file 12887_2024_4902_MOESM1_ESM.docx]

**Supplemental Table 1.** Baseline characteristics of no-IBD patients with *H. pylori* infection.

| **Total healthy controls with *H. pylori* infection** | 18 patients |
| --- | --- |
| Female | 8 (44.4) |
| Median age, years (range) | 13 (7-17) |
| **Gastric histology** | |
| *H. pylori*-related antral gastritis | 13 (72.2) |
| *H. pylori*-related corpus gastritis | 2 (11.1) |
| *H. pylori*-related pangastritis | 3 (16.7) |
| **Symptoms and signs for undergoing gastroscopy** | |
| Anemia | 0 |
| Celiac disease | 0 |
| Familiarity for celiac disease | 0 |
| Suspicion of celiac disease | 1 (5.6) |
| Recurrent abdominal pain | 0 |
| Diarrhea | 0 |
| Dysphagia | 1 (5.6) |
| Dyspepsia/epigastric pain | 8 (44.4) |
| Symptoms of gastroesophageal reflux | 1 (5.6) |
| Melena | 0 |
| Failure to thrive | 0 |
| Suspicion of eosinophilic esophagitis | 0 |
| Suspicion of *H. pylori* related gastritis | 5 (27.7) |
| Suspicion of IBD | 1 (5.6) |
| Constipation | 0 |
| Vomit | 1 (5.6) |
