# Supplementary material for: Helicobacter Pylori infection in children with inflammatory bowel disease: a prospective multicenter study
Source: BMC Pediatr. 2024 Jun 29;24:417. doi: 10.1186/s12887-024-04902-z (PMC11218114; doi:10.1186/s12887-024-04902-z)
Supplement: Supplementary file 3 — Additional file 3: Supplemental Table 3. Case–control model-based comparison between inflammatory bowel diseases (IBD) patients with H. pylori infection (cases) and without H. pylori infection (controls) at follow-up. Cases and controls were matched for age at diagnosis (± 2 years), gender, and type of IBD (Crohn’s disease or ulcerative colitis). [file 12887_2024_4902_MOESM3_ESM.docx]

**Supplemental Table 3.** Case-control model-based comparison between inflammatory bowel diseases (IBD) patients with *H. pylori* infection (cases) and without *H. pylori* infection (controls) at follow-up. Cases and controls were matched for age at diagnosis (±2 years), gender, and type of IBD (Crohn’s disease or ulcerative colitis).

|  | **IBD patients with *H. pylori* infection**  **n= 7** | **IBD patients without *H. pylor*i infection**  **n=21** | ***p*** |
| --- | --- | --- | --- |
| Female | 2 (28.6) | 6 (28.6) | Matched |
| Median age, years (range) | 14 (12-16) | 14 (12-16) | Matched |
| **Ulcerative colitis** | 4 (57.1) | 12 (57.1) | Matched |
| PUCAI 0 - 35 | 4 (100.0) | 11 (91.7) | 1.000 |
| PUCAI > 35 | 0 | 1 (8.3) | 1.000 |
| UCEIS 0 - 4 | 4 (100.0) | 11 (91.7) | 1.000 |
| UCEIS 5 - 8 | 0 | 1 (8.3) | 1.000 |
| **Crohn disease** | 3 (42.9) | 9 (42.9) | Matched |
| PCDAI 0 - 40 | 3 (100.0) | 9 (100.0) | 1.000 |
| PCDAI > 40 | 0 | 0 | 1.000 |
| SES-CD 0 - 6 | 3 (100.0) | 6 (66.7) | 0.5091 |
| SES-CD > 7 | 0 | 3 (33.3) | 0.5091 |

Data were expressed as number (percentage) of the total.

PUCAI: Pediatric Ulcerative Colitis Activity Index

UCEIS: Ulcerative Colitis Endoscopic Index of Severity

PCDAI: Pediatric Crohn’s Disease Activity Index

SES-CD: Simple Endoscopic Score for Crohn’s Disease
